# How Social Determinants of Health of Individuals Living or Working in U.S. Department of Veterans Affairs Home-Based Long-Term Care Programs in Puerto Rico Influenced Recovery after Hurricane Maria

**DOI:** 10.3390/ijerph192013243

**Published:** 2022-10-14

**Authors:** Leah M. Haverhals

**Affiliations:** 1 Denver-Seattle VA Center of Innovation for Value Driven & Veteran-Centric Care, Rocky Mountain Regional VA Medical Center at VA Eastern Colorado Health Care System, 1700 N. Wheeling St., Aurora, CO 80045, USA; leah.haverhals@va.gov; Tel.: +1-720-331-4176; 2 Department of Health Care Policy & Research, School of Medicine, University of Colorado Anschutz Medical Campus, Aurora, CO 80045, USA

**Keywords:** older adults, social determinants of health, disaster recovery, social capital, Puerto Rico, Hurricane Maria, home-based long-term care

## Abstract

In September 2017, Hurricane Maria devastated Puerto Rico, causing extensive infrastructure damage and a significant number of deaths. In the months and years since, recovery from Maria has been slow, hampered by delayed delivery of fiscal aid, corruption, economic hardships, and Puerto Rico’s colonial status. Simultaneously, Puerto Rico’s population is rapidly aging and hundreds of thousands of mostly younger Puerto Ricans are migrating out of Puerto Rico for more opportunities. Many Puerto Ricans who are older or disabled and need long-term care receive this care in home-based environments, as Puerto Rico has minimal institutionalized long-term care infrastructure and limited funding to expand it. The Department of Veterans Affairs (VA) offers several home-based long-term care options for Veterans in Puerto Rico. In this qualitative case study, veterans, VA staff, veterans’ caregivers, caregivers’ family members, and veterans’ family members receiving or involved with providing this care were interviewed regarding their experiences during and after Hurricane Maria. Specifically, this study highlights how social determinants of health of those residing in or involved with VA home-based long-term care programs influenced recovery from Hurricane Maria, and how findings can inform disaster recovery and provision of home-based long-term care going forward.

## 1. Introduction

In September 2017, Hurricane Maria devastated Puerto Rico, following Hurricane Irma which had struck a few weeks before. The reality of Puerto Rico’s colonial status [1] and economic fragility [2] has led to decades of many of its citizens living in poverty while navigating systemic social vulnerabilities amongst fragile infrastructure. These factors played an enormous role in Maria’s devastation of the island and its slow recovery. Further, the underwhelming responses from both Puerto Rico and United States (U.S.) governments complicated situations [3,4,5,6], particularly for the most vulnerable populations. These populations include older adults and people with disabilities who manage multiple chronic health conditions, many of whom receive home-based long-term care (LTC). The Puerto Rican population is also aging rapidly, with the number of residents aged 65 and older rising from 13% in 2010 to 22% in 2021 [7,8,9], leading to increases in demand for such care. Simultaneously, disasters, such as Hurricane Maria, are occurring with more regularity, ferocity, and unpredictability [10,11].

A physician working in Puerto Rico when Maria hit noted “the poor and vulnerable were disproportionately affected” by the hurricane [12] (p. 1801). Social vulnerability and social justice research has also found these populations suffer more when disasters strike [13]. In recent years, Puerto Rico’s economy has been negatively affected by hundreds of thousands of mostly young people leaving the island amidst dismal fiscal policies implemented by Puerto Rico and the U.S. [8,14]. Economic research [15] has shown that countries with struggling economies are at a significantly higher risk of climate-related disasters, which can increase the likelihood of social vulnerability factors rising—including increased mortality and morbidity. This is important to note as most Puerto Ricans in need of LTC receive it in home-based settings, as there is minimal institutionalized LTC infrastructure and insufficient funding to expand it [16].

In the first three to four days after Hurricane Maria, it was reported only three large hospitals were operational [12], one of which was the San Juan Veterans Affairs Medical Center (VAMC). The San Juan VAMC operated with backup generators [17] and worked with civilian hospitals post-Maria to assess damage and deploy mobile medical and counseling centers to veterans and the public [18]. Additionally, the U.S. Department of Veterans Affairs (VA) offers several long-term, home-based care options for veterans Past research has shown that “the long-term care sector often falls through the cracks” in both planning and responding to disasters [19] (p. 423). This manuscript describes how social determinants of health of those living in or involved with the Puerto Rico VA home-based LTC programs influenced these individuals’ recovery after Hurricane Maria. Social determinants of health are defined as social, economic, environmental, and political factors associated with health and health disparities [20]. This manuscript is part of a larger study studying VA and non-VA home-based LTC settings in Puerto Rico post-Hurricane Maria.

## 2. Materials and Methods

### 2.1. Study Design, Theoretical Approach, Participants, and Recruitment

#### 2.1.1. Study Design and Theoretical Approach

**Figure 1:**
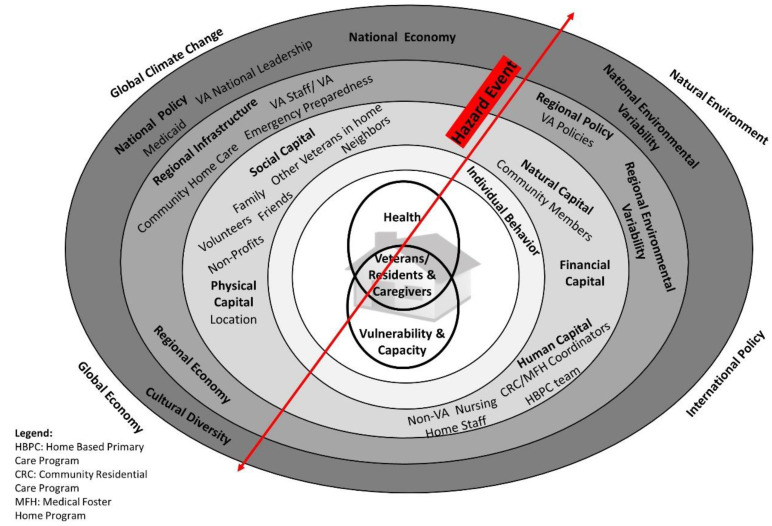
Conceptual Model of Factors Influencing Vulnerability and Health in Puerto Rico Home-Based Long-Term Care Settings Following Hurricane Maria. Model adapted from: Thomas, D., Scandlyn, J., Brett, J. & Oviatt, K. (2013). Health. In Thomas, D.S.K., Phillips, B.D., Lovekamp, W.E., & Fothergill, A. (Eds.), Social Vulnerability to Disasters, Second Edition (p. 244). CRC Press: Boca Raton, FL [22].

I designed this study using a qualitative case study design [21] of VA home-based LTC settings in Puerto Rico, post-Hurricane Maria. The study was guided by the theoretical underpinnings of the social vulnerability and health model [22] and Bourdieu’s writings on forms of capital [23,24], and there were several categories of individuals sampled for this project amongst different VA home-based LTC settings. These included those living in or involved with the Puerto Rico VA Medical Foster Home (MFH), Community Residential Care (CRC), and Home Based Primary Care (HBPC) programs. Figure 1 illustrates the adapted vulnerability and health model, which is an extension of the health ecology model and considers social determinants of health. An ecological model of health stresses the relationship of multiple levels of several factors—or social determinants of health—influencing health, and focusing on relationships between these factors. These include social, economic, environmental factors, and health disparities. The hazard or disaster event cuts through the various rings of the model.

#### 2.1.2. VA Home-Based Long-Term Care Settings

Three VA home-based LTC settings were studied: the MFH program, HBPC program, and CRC program. The MFH program provides full-time LTC to medically complex veterans who meet nursing home eligibility in the homes of private caregivers, and these caregivers are recruited and screened by the VA [25]. Up to three veterans/residents can reside in one MFH, making them very family-like settings. Veterans residing in VA CRC programs are also often highly medically complex, have no in-home caregiver of their own, and cannot live alone. CRC is offered in a variety of locations, including Assisted Living Facilities, and personal, family, or group homes, all of which are inspected and approved by the VA; MFHs are one type of CRC [26]. VA HBPC programs deliver comprehensive primary care to veterans in their own homes, and veterans usually live within about a 50-mile radius of the VAMC. HBPC programs are composed of interdisciplinary staff that can include physicians, nurse practitioners, registered nurses (RNs), social workers, rehabilitation therapists, pharmacists, dieticians, and psychologists, among others [27]. While HBPC veterans choose to remain living in their homes, they are often bedridden, wheelchair-bound, or have limited mobility, and require oxygen or ventilators [28]. MFH veterans receive in-home care from HBPC teams. Table 1 describes the three different VA care programs. While not described in detail in this manuscript, non-VA LTC settings in Puerto Rico have many similarities to VA settings. Most do not house a large number of residents, feel less institutionalized than nursing homes on the U.S. mainland, and are often small, family-run homes with about 10–20 residents.

#### 2.1.3. Participant Types

Types of participants in the study included: VA staff who operated day-to-day operations of VA home-based LTC programs in Puerto Rico; VA Geriatrics and Extended Care national leadership who provide oversight to these programs; MFH and CRC caregivers in Puerto Rico; caregivers’ family members; veterans in Puerto Rico VA home-based LTC programs; and families of veterans. The social vulnerability and health model informed sampling decisions in order to capture experiences of individuals in various rings of the model. This allowed for gathering trustworthy data [29] from multiple perspectives to better describe the influence Hurricane Maria had on individuals’ social determinants of health.

#### 2.1.4. Recruitment

I recruited participants primarily using snowball sampling techniques, where one participant recommends others they know to participate [30] and also distributed a recruitment flyer/study information sheet to interested participants. Prior to arriving in Puerto Rico, I invited leadership of VA LTC programs at the San Juan VAMC to participate in the study by email. They indicated interest and invited me to meet with members of two HBPC teams to explain the study and invite them to participate. Similarly, I first contacted two MFH Coordinators, one of whom also served as the CRC Coordinator, by email prior to arriving in San Juan, to request interviews and explain the study. They both agreed, and recommended potential participants who might be willing to participate in the study (i.e., caregivers, veterans, family members of veterans, and caregivers’ family members). Therefore, the sample was also a purposive (participants were recruited from the aforementioned groups) and a convenience sample (participants were willing and available to participate) [31]. Individuals serving in national VA leadership roles were invited by email to participate in interviews. No incentives were offered to participants.

### 2.2. Data Collection

I created four semi-structured interview guides, specific to participant type, and two were translated to Spanish. Interview guides were designed with grounded follow-up probes to encourage participants to expand on responses [32]. The VA staff interview guide is included as Supplementary Materials. I collected data in Puerto Rico from 27 January–14 March 2019, with interviews taking place in-person at VA clinics, in MFHs, and in CRC facilities. Following interviews, I asked participants to complete optional demographic questions. A research assistant fluent in Spanish and I conducted N = 44 interviews in either English or Spanish and we conducted N = 8 home visits to MFHs and CRCs. To ensure trustworthiness of the data, I was present during all interviews conduct in Spanish. As I have a working knowledge of Spanish, I asked clarifying questions as needed. Participants included: VA MFH or CRC caregivers (*n* = 10), VA staff (*n* = 15), family members of VA caregivers (*n* = 2), family member of veteran (*n* = 1), MFH veterans (*n* = 4), CRC veterans (*n* = 8), and VA national leadership (*n* = 4). Table 2 describes the different types and number of participants. Three national VA leadership interviews took place by phone and all interviews were completed in May 2019.

As data collection began 16 months post-Hurricane Maria, care was taken to account for recall bias among participants when asking questions about situations regarding their experiences, especially before and during Hurricane Maria. Many participants shared photos of damage following Hurricane Maria, which primed their memories. All interviews were audio recorded via a VA-approved digital recorder and with the agreement of participants, and participants were assured their responses were confidential. Interviews ranged from 12 to 75 min. Interviews were transcribed verbatim, and two research assistants fluent in Spanish translated interview recordings from Spanish to English.

### 2.3. Data Analysis

I applied an inductive and deductive approach to thematic qualitative data analysis [29,33,34]. Interview data were listened to and read through following transcription, and interview data analyzed using the qualitative data analysis software Atlas.ti [35]. I acted as sole coder and contacted the research assistant who conducted interviews in Spanish periodically to assure credibility and trustworthiness of the coding process [29,36]. Deductive codes were generated a priori based on the interview guides and initially applied as a primary coding structure. Inductive codes were created in vivo, emerging from the data, to identify relevant constructs that a priori codes did not capture. Analytic memos were created while coding to record thoughts on the analytic process and document early emergent themes. As coding commenced, I created categories to organize the data and subsequently queried data to determine main themes to answer the research question of how social determinants of health (social, economic, environmental, political, and health disparities) of those living in or involved with the Puerto Rico VA home-based long-term care programs influenced their recovery after Hurricane Maria.

## 3. Results

Five themes emerged reflecting how social determinants of health influenced recovery from Hurricane Maria, specific to the VA home-based LTC programs. These included: (1) economic instability, poverty, and political corruption in Puerto Rico; (2) various elements of social support, including: reliance on neighbors and volunteers to clear roads, provide food, repair electricity safely, and boost morale; (3) distrust of the government due to Puerto Rico and U.S. governments’ mishandling of recovery efforts; (4) social support networks between caregivers, veterans, and veterans’ families, (5) and VA staff’s teamwork and collaboration to support veterans. Overall, examples of how these social determinants of health related to recovery from Maria were exhibited in a multitude of ways by participants, some positively and others negatively. These findings reflect almost all elements of the vulnerability and health model, including social, cultural, physical, financial, human, and natural capital; infrastructure issues; local, regional, and national policy influence; economic factors; and environmental issues.

### 3.1. Economic Instability, Poverty, and Political Issues in Puerto Rico

Puerto Rico’s economy has been unstable for nearly two decades, and its status as a U.S. territory does not provide Puerto Ricans, who are U.S. citizens, full rights compared to citizens living in U.S. states. One HBPC staff member interviewed felt that recovery efforts related to Hurricane Maria were more difficult due to economic hardships, and because young professionals have been leaving Puerto Rico for many years to escape the economic uncertainty and pursue more lucrative job opportunities on the U.S. mainland. Many times, younger Puerto Ricans leave older family members behind. “The recovery after the Hurricane, it was a hard process and it’s not just this [physical clean-up/recovery], it’s political issues… I mean we’ll see what happens next, but at least I’m glad most of the patients are OK” (HBPC Dietician, 107). He continued on, and without knowing it at the time, foreshadowing the historic protests that erupted in Puerto Rico in July 2019, noting that:

“*The big problem that we have as a country right now is the political problem. It’s the economy and all like, the loss that is the Gen X [speaking of young professionals leaving Puerto Rico for the mainland]. They [the U.S. government] don’t help us, you know? The economy, we could be better if we don’t have that problem…We are in a situation, that I was hearing in the news that it’s true. Like we don’t know if within the next year it’s going to be maybe worse [the economy*].”

(HBPC Dietician, 107)

Another political issue mentioned by a few participants involved corruption in the Puerto Rico government, which they felt negatively influenced recovery efforts and resource distribution. One VA staff member noted that the police in Puerto Rico were under a “*big crisis*.” This was because the government did not have enough funds to pay the police, and they were “*getting retired massively*” (HBPC Occupational Therapist, 110). She also felt that the lack of police presence influenced recovery post-Maria, as many people felt unsafe being in the streets if they needed to travel or were trying to get food or water, especially because there were many desperate people who were struggling after Maria, which stoked people’s fears.

A few VA employees also discussed volunteering outside of the VA in the community post-Maria to assist with recovery efforts, and that areas with higher poverty experienced much more dire situations compared to those living in more affluent municipalities and neighborhoods. This included lacking needed resources and medications. One VA employee described such differences between veterans she cared for who were high needs and had to come to the VA hospital appointments.

*“I’d see one patient and he would be like, ‘I live in Condado [busy, touristy neighborhood in San Juan]. And my cistern is broken. And I’m gonna get a new one.’ And we’d talk about that. And the next patient, would walk in and be like, “Yeah, I lost my new house. I’m living with my daughter” in some other very far campo [countryside] city or town. ‘And we don’t have water and we don’t have electricity.’ And, just the differences, from literally one patient leaving and the next coming in. We’re just like, ‘Okay, we’re all on the same island. You’re all veterans.’ And yeah, that was, that was really heartbreaking because, so many of them don’t live in affluent parts of Puerto Rico. Besides San Juan, Puerto Rico’s, it’s a lot of poverty. And, it’s seeing them and knowing...oh the most heartbreaking ones were when like the kids lived in the States, and you know, these people are like, 90 years old and living alone*.”

(VA Pharmacist, 158)

Other participants also noticed stark differences between rural and urban dwelling veterans, usually with rural veterans experiencing more barriers to recovery. They noted that veterans with family living on the U.S. mainland often had just as difficult time post-Maria, not knowing how their loved ones had fared. One CRC caregiver noted,

“*I had patients whose families were in the United States. There was no communication. I didn’t have a [cell phone] signal. I’d go down to Caguas [town near her home] and there was signal... they must be worried about their family member and I can’t communicate with them. I couldn’t find the way until one day, being in line at Banco Popular, [the] phone started ringing. When I got home and heard all the messages from the families, I called them... they were calm, but they wanted to know, they wanted to be sure [the veterans were OK] and were willing to send whatever you needed. The families helped out a lot. They gave us money for gasoline. Their support was also there*.”

(CRC Caregiver, 137)

Interestingly, one HBPC staff member thought some of her veterans who were very poor would be struggling more after Maria, but because of their preparedness efforts and the strength of their communities, this was not the case. “*I was surprised. Because, for example, [with] some humble, you know, like really poor patients that we have, I thought that we were going to be receiving lots of complaints, and they were managing the emergency so well*”.

(HBPC Occupational Therapist, 110)

### 3.2. Social Supports’ Role in Recovery

Such examples of how some poorer veterans fared better than expected due to help from their communities, as well as how some veterans’ families provided extra assistance post-Maria, highlighted ways social support played a role in Hurricane Maria recovery efforts.

#### 3.2.1. Strength of Communities and Reliance on Neighbors

Participants relayed several stories of neighbors and communities banding together to help one another in the wake of Maria’s destruction. One HBPC dietician noted that “*Here in Puerto Rico, many neighbors are like family. And we see that for older people or older adults. They really, neighbors are really taking attention to those people*” (HBPC Dietician, 113). A specific example shared was of a few neighbors who owned a workshop on the same block as an MFH and provided the MFH with electricity from their workshop’s generator. These neighbors also figured out how to take electricity from electric poles and create some light for homes in the neighborhood. The MFH caregiver described their help as follows:

“*They would say, ‘Go to Kmart and purchase three tension wires of 100 or 250.’ And they would put them [lights] up from… all the way up there [pointing up high]… At night everyone had their lights on, but in the morning around 5 we had to disconnect it. I lasted about a month and a half with power from the streets, but in the day, I had to turn on the generator. So, I used to do the laundry at night. I would rinse off the urine off of their [her residents’] clothes and I would leave it there. At night I would put them in the washer and plugged it to the generator. But in the morning, we had to disconnect it. It was abusive because there was light everywhere else but in the houses [after they disconnected it]. At night you would pass somewhere and think that there was light, but the houses were in the dark. We had a very, very hard time*.”

(MFH Caregiver, 133)

Another MFH wife and husband caregiving team, who had been caring for a MFH veteran receiving hospice during Maria, shared many stories of helping neighbors, and vice versa, post-Maria. This included how they and their neighbors cleared streets of debris with axes and prepared food and meals for their neighbors.

“*It was something that, even though we passed through that [tough situation] during the hurricane, in the middle of that battle we learned something from it, because we spent time with the neighbors. I knew to take them ice. I knew how to tell them… so I could give them some ice. You learn a lot from those things.”*

(MFH Caregiver, 114)

Her husband added that “It was very difficult because the recovery, there was a lot of efforts. There were a lot of effort to help us recover. To this hour we are still lifting ourselves up. All the areas aren’t at 100% yet” (MFH Caregiver, 115). An HBPC physician echoed this story, stating in rural areas neighbors or relatives of veterans cleared the streets, and that by the weekend after Maria most of the neighbors had worked enough so the streets around her own neighborhood were passable:

“*They [neighbors] did all the jobs because… the government agencies were overwhelmed with work. So, they [neighbors] do a lot of the work. Even in my neighborhood… because we didn’t have any other assistance. So, most of the neighbors pick up the trees and all that stuff, so if there was an emergency, we can get out*.”

(HBPC Physician, 118)

An HBPC nurse also had similar experiences with the community banding together because they felt there was no other option.

“*That is how it was done [the community working together]. Mainly in the countryside. In the countryside, if I want to get out of the house, and I have a tree trunk, well the community would go, and the ones that had a chain saw would go and cut down the tree trunk, so they could come out. Otherwise they couldn’t come out. They couldn’t wait for the government … they had to go out shopping and see what they could find*.”

(HBPC Nurse, 143)

Many other VA staff members shared that some of the neighbors of veterans would stand in line for them for gas and water. “They have a lot of support from their relatives and neighbors. We have to do it, there’s no other way. It’s the only way to survive. We were in survival mode, literally” (HBPC Physician, 118). Others had stories of neighbors sharing food and meals, even if they did not know those neighbors well, but to make sure they were managing after the hurricane. One CRC Caregiver noted that he had an excess of water and would donate it to other non-VA LTC homes in need.

“*I had a lot of water. I had to donate water to other homes. Because they [local government] gave me a lot of water. I shared with other homes that were in a bit of a crisis. We didn’t communicate a lot, but we came together. I’d supply things that some didn’t have. After the hurricane, I had to donate to a home because they were not prepared*.”

(CRC Caregiver, 154)

A VA pharmacist echoed this sentiment, about neighbors coming together to help each other.

“*I think it just really speaks to how Puerto Ricans are as people. They’re just so loving and caring. And that was the number one feeling I felt after the storm, like everyone was just worried about everybody else and wanted to make sure people were okay, and reached out to strangers, and I think that was a really strong feeling afterwards.*.”

(VA Pharmacist, 158)

Neighbors of HBPC veterans assured HBPC staff they would continue to check on veterans after HBPC left. Examples of assistance between HBPC, CRC, and MFH settings and their neighbors included running extension cords from neighbors’ houses with generators to veterans’ homes who did not have one in order to power their refrigerators or fans. Neighbors would also swap resources, for example some would share their generator in exchange for cold water or ice. These types of exchanges especially helped HBPC familial caregivers caring for bedbound veterans. One HBPC dietician shared:

“*There was like this huge movement I would say, like a lot of people trying to help you show them a better connection. Just a lot of people that say ‘I make a lot of friends making the line for gas’… and like also the culture thing; like we talk a lot. We as Latinos we are more friendly… So that helped a lot because people try to help each other*.”

(HBPC Dietician, 107)

An HBPC physical therapist commented that “*Oh My God, Puerto Ricans give it here*.” Explaining what she meant, she noted:

“*I think that the reason that the people could survive this after [Maria]… was our neighbors. Just the people that were living here, got so much united to help. But in those first days, if it wasn’t for the, for us just to start opening roads. Or helping our neighbors, when you go, when you went to the houses everyone [said] “Well, my neighbor came, and she had a power generator and she gave me a line and I connected.” I saw a lot of that. I saw a lot of neighbors helping impaired elderly or people that have impairment or needed electricity. I have a, I had a very beautiful story of our patient that she [her neighbor] gave her the other line, so she could move the bed at night [to manually adjust a bed for a bedbound veteran]...a lot of Puerto Ricans giving food… water, that was very, very helpful for us*.”

(HBPC Physical Therapist, 108)

A CRC veteran summed up the social support amidst the recovery as “You have to have a lot of faith in God. It’s great to be surrounded by people that appreciate and love you” (CRC Veteran, 135).

#### 3.2.2. Working Together to Repair Electricity, Clear Roads

As noted previously, Maria’s severity was devastating to the island, unlike anything most people had ever experienced in their lifetimes. One brother of a CRC veteran, who was also a veteran himself, noted the extent of the devastation of nature affected many people’s health and well-being.

“*Everything in the country was devastated. Especially [for] the people that weren’t aware of the warnings. They stayed where they were, and many were picked up along with their houses… Many people, they don’t listen to the warnings, they don’t even realize that their houses are not going to withstand the winds, the rain at that speed. They don’t understand. Some of us, we got together with our family and we got together in the best house to care for each other. The majority of people here, I don’t know if they were veterans or not, but it was deadly, terrible. The streets, the buildings, the people became sick. They had not been sick before, but they became depressed.*.”

(Family member of CRC Veteran, 146)

Keeping the overwhelming destruction of Maria in mind, participants also noted various factors that made life feel as if it were returning to normal in the months and year following Maria. For example, once roads began to clear, big trucks more regularly passed by on the roads, and when electricity returned, that helped immensely. However, participants described the instability of the electric grid as the major problem post-Maria, which also had been unstable prior to Hurricanes Irma and Maria.

One CRC caregiver shared they organized their neighbors to work together to improve the electricity issue in their neighborhood. They shared that neighbors worked five days in the rain and many united to eventually restore power to parts of their neighborhood themselves, because it took the electrical company four months to visit their neighborhood. Once restored, they had a huge neighborhood party “*like New Year’s Eve.*”

Caregiver 1: “*We had to take the initiative ... the neighbors sought out how we were to cooperate, we looked for people with understanding of electrical energy. Because with the generators, the gases, the noise. Economically speaking the generators… You couldn’t have them running 24 h because they could get damaged. I had to buy another generator to be able to use it*.”

Caregiver 2: “*One at night and one during the day. To be able to rotate them*.”

Caregiver 1: “*And they’d [veterans] want to watch their TV. And to keep them [veterans] distracted [post-Maria].*”

(CRC Caregivers, 137 and 138)

One CRC caregiver caring for five high-needs veterans in the countryside also shared a similar story related to electricity restoration. She said the only reason her electricity was initially restored was because of neighbors working together, because the electric companies were not focused on restoring electricity to individual properties. She added that she would have had to pay the electrical company to come and restore her electricity, if she wanted it to be a priority.

“*It was because of people, neighbors, who were able to pick up those lines, I don’t even know how they did it. They brought them together, of course, they are expert electricians. But they don’t work with the electrical grid [i.e., with the power companies]. They knew that there were people being cared for here. They told me, ‘Don't worry. We are electrical experts; we are going to make it so the electricity [returns].’ So, they... that cable, they raised it and they gave us electricity*.”

(CRC Caregiver, 147).

Another CRC caregiver caring for the 15 veterans in a rural area shared a story of how he and his neighbors worked together to clear the road near their homes that was obstructed from fallen trees post-Maria. The road in front of this CRC was the only route in or out in the neighborhood, and also eventually dead-ended, so it was critical to clear it. This caregiver had purchased a chainsaw in advance of the storm, anticipating potential need because of the high likelihood of fallen trees. Another neighbor also had an additional chainsaw. The caregiver shared:

“*We organized and the day after the hurricane, around 1 in the afternoon, we all worked to clear the road…If I am in a situation and I need that road to be cleared, there is no other way; there is no other road that I can take. It’s a street with a dead end. Thanks to God, that day we all cleaned up because no one could go out. We are about 12 houses, we were stuck, we couldn’t go out. We all cleaned it up, we opened the road and were able to get through*.”

(CRC Son/Caregiver, 145)

Yet another CRC caregiver also shared a similar story of supportive neighbors helping to clear her steep, rural driveway the second day after Maria hit.

“*What happens is that we have a nice community. Because on the second day [post-Maria], there came three gentlemen looking for where the trees had fallen, with a machine, all of them with machines, to cut down the trees. They came in, and we went out running, my daughter and I, we went all the way down there. ‘Look at this, it has to be moved, because we can’t get through.’ In case there is an emergency… So, they came and cut down those trees. The cable lines fell down. They took out the cables. So, they allowed for cars to come up and go down… That was accomplished in this part right here, because of their help. Then they went on to help the neighbor and they took out all the trees that were in the middle there. And they went on helping others. It was a blessing from our Lord that there are people in the community who see the need that there is and they themselves help out.*” (CRC Caregiver, 147).

Another CRC caregiver caring for veterans and non-veterans in a rural area noted that her husband and neighbors began clearing the road in and out of their CRC, which also had only one way in and out. After four days, employees from the local government came to assist. She noted that after Irma, the municipality did not come, and it was only her husband and the neighbors who worked to clear the road.

“*With Maria, the neighbors were out with their saws and the machetes to clear the trees. You had to get creative. Take the posts that were lying there and move them... until the road started clearing up. The neighbors worked together until the fourth day. [The] fifth day, the municipality came. One of the families knew the mayor, so the mayor sent the digger*.”

(CRC Caregiver, 154)

### 3.3. Mishandling of Recovery by Puerto Rico’s Government and the Federal Government

Many participants felt that both the Puerto Rico and U.S. government mishandled recovery efforts. One nurse stated a common theme that recovery efforts were chaotic. “*There was no organization whatsoever. That is my impression. There was no organization. Nothing. Very difficult*” (HBPC Nurse, 143). One CRC caregiver felt the support and response from the federal and Puerto Rico’s governments was inept, stating “*That was another disaster. They did not help out at all. It’s like I told you, they require a lot of things from you but in the moment that something happens, there are no answers from them*” (CRC Caregiver, 144). Another issue some participants raised related to the Jones Act, which states that goods shipped between any two ports in the United States must be carried on ships that are American built, owned, and operated by U.S. citizens/permanent residents. This affected Puerto Rico, especially following Maria, because many goods had to pass through Jacksonville, Florida before going to the island; they could not go directly to Puerto Rico (while the Jones Act was temporarily lifted for 10 days due to Maria, from 28 September 2017–8 October 2017, it was then reinstated). This led to challenges including that many resources sent to Puerto Rico, like bottled water, were not distributed on time and went bad because of delays.

Additionally, there was distrust of the U.S. Federal Emergency Management Association (FEMA) due to lack of communication from the government about how to access FEMA resources. Participants shared that where and when to access FEMA resources was largely spread by word of mouth. During the time of data collection, blue tarps, acting as temporary roofs for roofs the hurricane damaged or tore completely off, remained on many homes, which participants attributed to lack of FEMA assistance. These and other similar situations led people to think it was easier to access resources from community organizations and non-profits than from the federal or Puerto Rican government.

“*I think [there’s] more trust between people. I think, not that Puerto Ricans don’t trust the federal government, but I think they’re more likely to trust their neighbors, and people they know, and I think FEMA also dropped the ball. And then people associated all federal help with just, a mess or false promises, or ‘It’s going to take forever’, or ‘They’re not gonna actually pay me what they said they’re gonna pay me’, or ‘They’re not gonna actually give me a roof’. I think there’s just so many, like, one thing after another kept falling through, and people were like, ‘Okay, well, I need to find some sort of other help, because this is not gonna work’*.”

(VA Pharmacist, 158)

#### Appreciation of Aid when It Did Arrive, Especially from Local Municipalities

Despite these challenges, there were many examples of appreciation of assistance from military aid and other aid organizations. One program coordinator shared an example of a CRC veteran who advocated for assistance for he and his fellow CRC veterans when he happened to be in the same area as some individuals from Puerto Rico’s power company, and coincidentally, some military staff with connections to resources.

“*I have a story of one veteran who was living in a CRC foster home… that didn’t have power, but the caregiver had a power generator, and then the energy company here in Puerto Rico was doing some reparations in the community and the veteran was going to the people saying ‘Hey! I live in a foster home where all the residents are veterans!’ And there were some military staff with them [the electric company] and the military said, ‘Oh, you are a Veteran?’ ‘Yes, I am a Veteran and I live there, and we have been without power since many months ago’. And the military people make the connection with the emergency staff… and because of the military that foster home have power that day*.”

(Program Coordinator, 104)

Another HBPC staff member noted that some of their HBPC veterans, and also herself and her family, had solid support from their local municipality in the form of water and military rations. Others mentioned receiving a lot of assistance from religious volunteers and churches.

A CRC caregiver shared that more aid from the Puerto Rico and federal government would have been the “*most useful*” towards recovery, as well as more assistance from the VA. However, he and his wife were aware since their CRC was in such a rural area that this proved more difficult. They were able to receive some assistance from their municipal government, which they appreciated.

“*They [the VA] were always willing [to help] but obviously, no one could come all the way out here... But at least the local government was able to help more. They were more on top of things. For them [the Puerto Rico and federal governments], politics are always first, and these things are not... We did not see that help from the government. That commitment to help us without having to ask for it. We had to ask for help and they gave it to us within their ability. They’d help us, but not because it was born out of them [i.e., because they volunteered assistance]. Or because they knew there was a home here*.”

(CRC Caregiver, 138)

Additionally, some HBPC staff members made efforts to explain to veterans’ relatives how to access resources from the municipal governments. For example, municipalities could provide the veterans with gas for generators daily, if the generators had been provided to veterans or their caregivers by FEMA. Many families were not aware of this system, so HBPC staff helped to inform them and bridge this communication gap.

“*We informed them, ‘You have to go… there is an office in every municipality that were working with that.’ And we just orient their relatives to go to that office, ask for them because we know every municipality have it. And some of them after we orient them, they went*.”

(HBPC Physician, 118)

One participant in VA national leadership noted that, “*We [the VA] have to do a better job of how we get the supplies to those homebound people*” (VA Leadership, 163). She was speaking about how non-profit organizations like the Red Cross set up stations for people to come and pick up resources, supplies, and water. Of course, homebound veterans cannot often access these resources themselves easily, due to mobility issues, disabilities, and lack of transportation.

“*So, what do we do? We [the VA] do a really good job of knowing where our patients are, but we can’t get them supplies. They can’t go out and get water. They can’t go out and get to the grocery store. We have to do a better job of, once we identify where they are: Can they actually get supplies? And then us as the VA, connecting with the Red Cross or connecting with the community to be able to get them the resources that they need*.”

(VA National Leadership, 163)

She stressed the importance of partnering with such volunteer or non-profit agencies, nothing the VA and its staff, “We’re not emergency responders, and we’re not that community agency. We don’t have the supplies and we can’t go stand in line for them” (VA Leadership, 163). She continued to say that with home-based LTC programs, disaster preparedness was a key role the VA could play due to the fact that the VA’s role is not as emergency responders. She added:

“*A lot of times, facilities will be like, ‘Oh, you’ve got a patient, we got to get their medications. Can you go deliver their medication?’ Well, the answer’s no. We don’t want our HBPC staff out during a storm. I mean, it’s just not safe. So, our role is really more to be able to communicate with the command centers, track our location and movements of our patients, what their needs are, and you know, really where they are, and how to get to them after the storm passes, and then track them. You know, after the storm passes and know where they are*.”

(VA Leadership, 163)

### 3.4. How Social Support Networks between Caregivers, Veterans, and Veterans’ Families Proved Critical to Health and Recovery

Older individuals acting as caregivers for older veterans was common in Puerto Rico. An HBPC physician commented on this reality, relating it to family structure, stating that many veterans in Puerto Rico had their families very young. “*The older daughter that is taking care of them is in their 70s or even almost in the 80s, too. So, it’s like old people taking care of old people*” (HBPC Physician, 118). This then became an issue of how the caregivers’ health was holding up and became an extra issue for VA staff to manage related to veterans and their caregivers.

“*Sometimes here in Puerto Rico, it’s pretty strong [i.e., common] to stay with the relatives at home and take [care of] them. It’s like, culturally [the] way to take care of elders. And sometimes they [older caregivers] postpone their health care needs and doesn’t place the veteran in a nursing home or long term care facility... And I say, ‘If you don’t take care of yourself, what will happen with the veteran, right? They will have to be placed in some, in some institutions… do your stuff now and then you can enjoy your time with veteran’*.”

(HBPC Physician, 118)

There were many examples of caregiver dedication to veterans during recovery from Maria, especially in MFHs and CRCs. For example, the owner and main caregiver of one CRC with 15 veterans during Maria was traveling outside of Puerto Rico with three of his veterans when Maria hit. Thus, one caregiver, who had worked in the CRC for 11 years, stayed on for three days post-Maria, caring for the veterans around-the-clock, not returning to her own home and instead cooking for them, providing for their daily needs, and attempting to ease any anxiety they were experiencing. Once the CRC owner/caregiver could return to Puerto Rico, he made sure to support his staff, providing them food to take home to their families and working to create a strong sense of community and family-like atmosphere, all year round.

“*It is very important that our patients have recreational time. To be able to get out and do something different. We have outings, visits to the beach. Simple things. My mother sometimes will start a barbecue and makes them burgers as if we were at the beach but in the home. They have a nice time, singing. We put on music, the microphone. And we spend the day in our residence*.”

(CRC Son/Caregiver, 145)

Another CRC who cared for veterans and non-veterans also served as a refuge for some of their caregivers, especially since so many areas near their CRC were without water and power. This CRC had a generator and ample supply of water.

“*I had three employees [caregiving staff] who lost everything. The only thing left was the ground. So, what I did was tell them, ‘You can stay here.’ They didn’t have food... They didn’t all prepare at home, so there was no food [at their homes]. They didn’t have gas… There was no way of purchasing; whoever had not managed to purchase a gas stove, a gas tank, by then you couldn’t get one. People could not cook. Employees ate here. But at that time cooking was done for everyone... We united. I told them, ‘You can come at a certain time.’ And I’d give them food.*

(CRC Owner/Caregiver 153)

Another CRC caregiver noted that of the five veterans he and his wife cared for during Maria, four of them had family that would visit post-Maria, sometimes providing food and leaving extra money for more necessities to be shared with all the veterans there. Other veterans’ family members, however, would only come to pay for their veteran’s care, or not come at all. These two caregivers would creatively try and improve the family dynamics between veterans and their families in the challenging instances.

“*There are families that perhaps are not very close to them [the veterans], but as a home we try to promote closeness. We at times seek ways for them to be involved with them [Veterans]. We try to create a habit in them to come. We’ll ask for something for them, but it is just an excuse to get them to come here to see them*.”

(CRC Caregiver, 138)

Another CRC caregiver who cared for five veterans during Maria with her daughter had assured her veterans’ family members, in advance of Maria, that if there was something seriously wrong with the veterans’ health due to Maria, the caregiver and her staff would find a way to reach the family in person. Basically, if caregiving staff did not pay the family a visit post-Maria, they could rest assured the veteran was healthy post-hurricane. This caregiver noted she chose this approach “*Because you also have to give them [veterans’ families] peace of mind. You can’t have them stressed out. So, they can continue to function with their families and with their work [post-Maria]*” (CRC Caregiver, 147).

### 3.5. VA Staff Working Together Assisted in Recovery

Within a few days to one week post-Maria, VA staff went in pairs to all HBPC, CRCs, and MFHs across Puerto Rico to check on veterans and their caregivers, navigating perilous infrastructure and often without cell phone service. HBPC staff discussed how their own teamwork buoyed them through difficult times post-Maria and helped them in recovery. One HBPC nurse shared that what helped her the most was:

*“Other coworkers. The help from amongst us. We helped each other so we wouldn’t go around alone. If something should happen while in the car, there were no phones. If we had a flat tire, there was no way to get around that. What helped us the most was the support that we had for each other. We’d never go out alone, we always [were] with somebody else*.”

(HBPC Nurse, 143)

Another example of this teamwork and support was that VA staff operated a VA clinic out of a tent erected in the parking lot during Veterans’ Day in November 2017, and they decided to celebrate Veterans’ Day in the tent. “*It was something really, really beautiful*” (HBPC Dietician, 113). Afterwards, the medical director of the clinic took the flags that had been flying during Maria and had them framed to hang in the clinic once it reopened, where they still hang.

Another nurse shared that what helped most in recovery was “Humanity, communication, teamwork. Because without teamwork, nobody can survive; not a way for other people to, to get our needs complete, you know? Always get help from others” (HBPC Nurse 112). She continued by sharing examples including helping veterans access resources, like food and water, and making arrangements for HBPC veterans who may have needed to move out of their own homes post-Maria to private, nursing-home type settings, or to MFHs or CRCs. VA MFH and CRC staff also decided to hold a Christmas party for their veterans in December 2017 to help ease any anxiety the veterans were experiencing post-Maria, as at this time the electricity was still out in most facilities or run temporarily by generators. VA staff recognized MFH and CRC caregivers at the party, noting:

“*The immense work they [the caregivers] did [before, during, and after Maria]. They maintained the same quality of life for our Veterans; the same as before. They maintained that same quality… We danced, and we had a Christmas party. It is a way to forget and to distract them from that adversity of that situation, that anxiety of not having electricity*.”

(CRC Social Worker, 157)

One HBPC nurse noted that in the wake of Maria, as after other disasters, it is of great importance that veterans and caregivers know VA staff is there for them. She shared:

“*I think the most important thing is the contact with the patient or family member, be it via phone or if you can, visit the home. Visiting is so important because this culture likes that, for it to be face-to-face, to see the professional. And to educate the patient in person. They are very grateful when we visit the home and continue to track the patient in whatever they need in that moment. Be it foot care, blood pressure, diabetes, high blood glucose, whatever their condition may be, they prefer that continued care is provided by visiting the home. That is what I think is the most important*.”

(HBPC Nurse, 143)

Additionally, many volunteers came from other VAs to assist in providing care for veterans across various service areas/departments in the main VA hospital and the clinics around the island, which lifted people’s spirits. The caring culture participants described as specific to Puerto Rico shone in VA clinic visits, too, not just by the veterans, but also by clinicians receiving heartfelt inquiries from veterans and veterans’ families. When asked about supporting caregivers and if family was often present with veterans at visits, one VA staff member noted:

“I feel like it’s kind of part of the culture here… like whenever they come in and they have appointments, it’s like it’s like a party. We talk to the daughter and then you hear about the granddaughter… I just feel like that [the] culture down here is very, like open and talkative and inclusive. So, but yes… everyone would want to tell their stories [post-Maria]. So, like, “’This is my experience. This is what happened. This is where I was. This was who was with me.’ And then also, their concern for us [VA staff] was, like, very something that really shocked me. Like here they are, these patients who had like, lost everything. And they’re like, ‘But where were you? Donde estaste aqui?’ [during Maria]… That was something that really stuck out to me. Like just how much they cared about us, was like, really touching.”

(VA Pharmacist, 158)

Interestingly, one CRC social worker noted that as time passed, even with Puerto Rico’s tightknit culture, camaraderie faded as time went by between VA teams and colleagues, and she felt in Puerto Rico overall. However, she said the teamwork and cooperation withstood the test of time within CRCs and MFHs, because of the tightly knit nature of the veterans and caregivers in these settings.

“*I sometimes think we need another hurricane to see if we can become even more sensitive. Because we were more sensitive for some time. You’d see everyone, very friendly. Everyone cooperating. I miss that. I miss that brotherhood that once the power and water returned, we are no longer a collective, we are individuals, we live on the individual level. The veterans of course have always had that. They treat each other like family*.”

(CRC Social Worker, 157)

## 4. Discussion

Hurricane Maria’s impact on Puerto Rico, and its citizens who still reside there and those who have joined the diaspora, will be decades long. Participants interviewed for this study marked time by “Before and After Maria”. Researchers have noted that Maria will become a “permanent marker” event which they will compare other disasters to, influence how they prepare for them, and influence their vulnerability [37]. Applying the vulnerability and health model in this study showed that certain social determinants of health negatively affected those living in or working with VA home-based LTC programs. These included delays in disaster response and mishandling of recovery efforts by the Puerto Rico and the U.S. federal government [38]. In turn, the model highlighted how individuals were positively built up by Puerto Ricans’ communal spirit and activation of their collective social support networks, drawing upon various sources of capital—social, cultural, human—reflected in the model [23]. For example, veterans in the VA programs were greatly supported during this time of crisis due to reliance on their networks and collaborative efforts of their caregivers, neighbors, VA staff, and families. This proved critical especially considering the underwhelming responses from governmental entities post-Maria.

Application of the vulnerability and health model proved especially useful when considering the implications of various contextual factors the model includes, such as Puerto Rico’s government’s policies, economic realities in Puerto Rico, high poverty rates, Puerto Rico’s colonial status, long-term health effects, community resilience and cooperation, environmental factors, and global climate change. Researchers have noted how devasting the environmental impacts were post-Maria, and how they were worse for the most vulnerable [39]. The U.S. government’s Heathy People 2030 objectives outline five social determinants of health domains: economic stability, education access and quality, health care access and quality, neighborhood and built environment, and social and community context [40]. Research has shown Maria negatively affected all these domains [11], and the findings from the current study support these findings. Other scholars have noted how Puerto Rico’s colonial status places it as a disadvantage related to many social determinants of health [41]. In fact, some have called its colonial status “the ultimate social determinant of health in Puerto Rico” [1] (p. 31). Economic woes [42], high poverty rates [41], subpar infrastructure [42], and mass outmigration [43] remain huge challenges, especially in the context of global climate change.

Historic protests in Puerto Rico in July 2019—fueled by years of corruption, the stagnant economy, anger over school closings on the island, and lack of hurricane relief aid—resulted in the ouster of the then governor, while bolstering Puerto Ricans’ spirits [44]. However, the reality of delayed Hurricane Maria fiscal aid remained. On 16 January 2020, USD8.2 billion more in aid for Hurricane Maria recovery was released by the federal government, on the same day that then-President Donald Trump declared a major disaster in Puerto Rico due to earthquakes earlier that month [45]. Before then, only USD1.5 billion of USD20 billion in appropriated aid had been distributed [46]. For context, Texas received USD141.8 million in funding nine days post-Hurricane Harvey in 2017 compared to Puerto Rico’s USD6.2 million nine days post-Maria, even though Puerto Rico’s needs were greater [6]. Other scholars have also criticized FEMA’s coordination of disaster efforts as problematic, preventing local agencies from prioritizing efforts [6,47]. Notably, many study participants shared similar frustrations that local non-profits and local agencies could not take charge in distributing aid and resources even though they worked with and alongside FEMA post-Maria. It remains to be seen how new recovery efforts will compare with the recent devastation Hurricane Fiona had on Puerto Rico, hitting on 18 September 2022 nearly five years to the day that Maria hit [48].

Stories shared in this study about people banding together to help one another post-Maria resemble those of others studying social capital in the wake of major disasters [49], who found that neighborhoods with higher levels of social capital focused more on rebuilding and recovery than those who did not, and were more successful in these efforts. In the current study, social capital at the neighborhood level proved critical, as evidenced from the many examples of clearing roads of debris, individuals fixing their own electrical poles, and sharing food with neighbors in need. It is important to consider how critical social capital at the neighborhood and community level was to individuals in these LTC settings post-Maria, while at the same time realizing that many people may have less social capital to drawn on.

For example, research has shown that perceived social capital has been shown to decrease amongst older adults in the context of disasters [50]. When this is the case, sometimes individuals can draw on other forms of capital. Post-Maria, some study participants accessed their cultural capital, such as when VA staff delivered needed resources to veterans and caregivers. Participants also drew on economic capital to access supplies and receive needed medical care. Such interplays of capital are common in the context of resilience and disaster recovery research [51]. However, the question remains of what to do for those who are older or disabled who do not have strong social support networks, may not have adequate economic capital, and cannot draw on cultural capital. This is especially critical due to Puerto Rico’s rapidly aging population [8], which will continue to pose challenges as there exists both a lack of LTC infrastructure and a lack of caregivers.

While caregivers and VA staff interviewed in this study cared greatly for their veteran patients, treating them as family, many underprepared for Maria themselves. They underestimated the potential of the hurricane, navigating a culture of consistently thinking hurricane warnings would be worse than the actual storm, as for decades such a powerful hurricane had not hit Puerto Rico [52]. Unsurprisingly, caregivers in MFHs and CRCs who lived in the same building or house as their veterans were very well prepared, while those who did not live directly with those they cared for were less prepared. This reality may continue to have consequences for people as disasters increase and more people need live-in caregivers but do not have them.

Finally, the issue of loss of human capital related to continued outmigration of Puerto Ricans largely to the U.S. mainland continues to influence Hurricane Maria recovery. Many who left years before sent resources back—including generators—to their loved ones in Puerto Rico, displaying the critical role of the diaspora in recovery [53]. Further, a few participants shared how some of their families living on the U.S. mainland were more distressed post-Maria, not knowing for weeks how their families in Puerto Rico fared, which has been reflected in other Hurricane Maria research [54]. This outmigration has negative implications for LTC in Puerto Rico. Some sources estimate that in the 10 years before Maria, 500,000 Puerto Ricans migrated to the U.S. mainland; estimates post-Maria were that over 200,000 residents left in the first 10 weeks alone, and many anticipated this trend to continue [53,54,55]. Several participants in this study shared their concerns about who would provide needed care for older adults in the future, as so many able-bodied, younger Puerto Ricans were leaving. This is occurring while older and disabled people remain in Puerto Rico, or return from the mainland to retire in Puerto Rico, continuing a cycle of circular migration [56,57].

## 5. Conclusions

While the resilience and collaborative nature of the Puerto Rican people proved instrumental in recovery post-Hurricane Maria, these attributes cannot be the only factors to rely on during times of major crisis. The electric grid must be stabilized, and there must be improved federal and Puerto Rican government response—and coordination with local governments and non-profits—especially as Hurricane Fiona caused considerable damage. Further, there has been little research to date on home-based LTC programs post-disaster. While more is needed, findings from this study can inform future recovery efforts, especially as this study showed that older veterans were well supported despite how some social determinants of health negatively affected recovery. Thus, VA programs can be leaders in supporting other, similar non-VA home-based LTC settings following inevitable future disasters. However, Puerto Rico’s colonial status and many social determinants of health challenges will continue to complicate disaster recovery unless they are more fully addressed.

## Figures and Tables

**Table 1:** Types of VA Home-Based Long-Term Care Programs.

	VA Medical Foster Home (MFH)	VA Community Residential Care (CRC)	VA Home Based Primary Care (HBPC)
Description of Long-Term Care Program (oversight, type of caregiver)	Up to three veterans/residents live in the private home of a non-VA caregiver, who is recruited and screened by the VA	Size can vary from small, family-sized homes to larger facilities. Can house larger number of veterans. Facilities are inspected and approved by the VA. Veterans have no family or caregiver of their own and receive daily care from CRC caregivers who are non-VA employees	Veterans usually live in their own homes, usually with a familial caregiver, or they have a caregiver who lives nearby, or veteran lives alone. Veterans are enrolled into HBPC program by the VA
Description of care provided/care needs	MFHs are a type of CRC, usually with more medically complex veterans than CRCs. Veterans receive care from HBPC interdisciplinary teams	Veterans often have mental health care/psychiatric care and medical needs. While intended for less medically complex veterans, many in CRCs are also wheelchair-bound and need more advanced care. Veterans go to the VA for medical care	Veterans receive longitudinal care in-home from VA HBPC interdisciplinary teams who partner with the veterans’ caregiver(s) (if veteran has a caregiver) to provide care

**Table 2:** Type and Number of Study Interview Participants.

Type of Participant	Number
VA MFH/CRC Caregivers	10
VA Staff	15
Family Members of VA Caregivers	2
Family member of Veteran	1
VA MFH Veterans	4
VA CRC Veterans	8
VA National Leadership	4
**TOTAL**	44

## Data Availability

As this study is a research study for the U.S. Department of Veterans Affairs the raw interview data cannot be shared publicly.

## Supplementary Materials

The following supporting information can be downloaded at: https://www.mdpi.com/article/10.3390/ijerph192013243/s1.
