# Supplementary material for: How Social Determinants of Health of Individuals Living or Working in U.S. Department of Veterans Affairs Home-Based Long-Term Care Programs in Puerto Rico Influenced Recovery after Hurricane Maria
Source: Int J Environ Res Public Health. 2022 Oct 14;19(20):13243. doi: 10.3390/ijerph192013243 (PMC9603221; doi:10.3390/ijerph192013243)
Supplement: Supplementary file 1 [file ijerph-19-13243-s001.zip › ijerph-1920272-supplementary.pdf]

Puerto Rico Hurricane Maria Study
<u>Interview Guide for VA STAFF</u>

- Researcher: ____
- Date: ______________________________ Start Time: __________End Time: ________
- Study ID: _________________

My name is [interviewer name] and helping me today are/is [additional team member(s)]. I am a researcher from the VA. For this IRB-approved research study I/we are interviewing individuals involved with the Puerto Rico VA **including VA staff in the Medical Foster Home program, Home-Based Primary Care program, community residential care programs, and other VA staff involved in emergency preparation and planning**. We hope to learn firsthand from you:
(1) about disaster plans in place before Hurricane Maria
(2) experiences implementing those plans
(3) your experience with Medical Foster Home Veterans and Caregivers during and after Hurricane Maria
(4) your experiences during and after Hurricane Maria
(5) and how lessons learned from the Hurricane have influenced policy going forward around preparing for disasters.

[Give participant Study Information Letter (which was also previously emailed) and Informed Consent Document. Explain Informed Consent Document, obtain signatures, give them a copy]

Your participation in this interview is voluntary, and you can choose to participate or not, or end the interview at any time. You can skip any questions you do not want to answer or do not know the answers to. Everything you share is confidential and you will not be identified in any reports, presentations, or publications. Your participation will last about 45-60 minutes for this interview.

To make sure we capture all the information you give me, I would like to record this interview. The audio-file for the recording will be stored in a restricted access research file folder on the VA intranet. Is this okay with you? **[Hit record button.]** Okay, to confirm, I'm starting the recording. Is this ok with you?

Grounded prompts: If responses are limited or require clarification, probes may be used to elicit more detailed responses. Probes should use words or phrases presented by the participant using one of the following formats:

1. ***What do you mean by ____________?***
2. ***Tell me more about ____________ ?***
3. ***Can you give me an example of ____________?***
4. ***Can you tell me about a time when ____________?***
5. ***Who____________?***
6. ***When____________?***
7. ***Where ____________?***

1. Tell me about your role at your VA.
   a. How long have you served in that role?
   b. Tell me about your previous experience providing home-based care or working with long-term care populations.

2. Tell me about taking care of Veterans during and since Hurricane Maria.
    a. How have caregivers/Veterans fared since the Hurricane?
        i. Specifically, how has Veterans' health been affected? (physical/mental/relationships?)
    b. How have you worked with/supported caregivers/Veterans/Veterans' families since Hurricane Maria?

3. Tell me about disaster plans or policies in place at your VA before Hurricane Maria.
    a. Specifically, what disaster plans were in place regarding home-based Veterans?
    b. How are these disaster plans the same or different for VA Medical Foster Homes, HBPC Veterans, or CRCs?
    c. Who is involved in creating these plans or policies?
    d. What training have you received around these plans or policies?

4. Please describe how you feel the disaster plans were implemented during and after Hurricane Maria.
    a. What went well?
    b. What were some of the challenges?
    c. What would you have liked to see improve?
    d. Have you experienced a disaster like this before? If so tell me about that experience.

5. Tell me more about your involvement with or knowledge of the VA Medical Foster Home program, HBPC program, or CRC program.
    a. What health issues do the Veterans you care for have?
    b. How were their health issues affected during and after the Hurricane?
    c. How did the Hurricane affect your ability to care for them?
    d. How did you communicate with Veterans and Caregivers during the Hurricane and after?

6. Tell me about the training provided to VA caregivers in disaster preparedness.
    a. What if any other resources related to disaster preparedness are caregivers provided from the VA?
    b. What if any additional disaster preparedness efforts are you aware of that caregivers have in place since Hurricane Maria?

7. Tell me about your experiences during and after Hurricane Maria.
    a. Biggest challenges?
    b. What has helped you the most during recovery from Hurricane Maria?
    c. What resources would have been helpful that you did not have?

8. Tell me about lessons learned from Hurricane Maria.
    a. How have these lessons informed policies going forward around preparing for and recovering from disasters?
        i. If they have not informed policies, how would you like them to?
    b. How have these lessons influenced caring for Veterans with long-term care needs, like those in the VA programs?
    c. What would you say are the <u>key ingredients</u> to successful disaster preparedness and recovery related to long-term care programs?
    d. If you could design the ideal disaster preparedness plan/policies, what would they look like?

9. What other things do you feel are important for me to understand about disaster preparedness or recovery when caring for who have long-term care needs?

10. Are there other things you feel are important for me to understand ***your experience*** implementing disaster preparedness plans and caring for Veterans who have long-term care needs?

11. If I have further questions follow-up questions would you be ok with me contacting you later?

12. Finally, is there anyone else you would recommend I talk to on this topic?

Thank you very much for your participation today!
